# The Recovery Rate in Mental Hospitals

**Published:** 1912-10-26

**Authors:** 


### The Hospital

The Workers' Newspaper of Administrative Medicine and Institutional Life, Administration, National Insurance and Health.

No. 1373, Vol. LIII. SATURDAY, OCTOBER 26, 1912. PRICE ONE PENNY.

### THE RECOVERY RATE IN MENTAL HOSPITALS.

Deviations from the straight and narrow pathway of truth have been classified in a way which is complimentary to the powers of imagination possessed by the statistician and the expert witness. In comparison with their finished productions the rude and simple attempts at falsehood of the ordinary man in the street are hardly to be reckoned as departures from strict veracity. When, however, the statistics alluded to are the work of the expert witness the summit of unreliability should, according to the freely expressed opinion of the legal profession, be reached. This may be said without in the least impugning the character of the experts assailed. It is, unfortunately, true that the most unreliable and dangerous figures may be the product of the labours of the most careful and painstaking compilers. Take, for example, the question of the influence of alcohol in producing mental disease. We should, and rightly, hesitate to place implicit reliance on the result of an inquiry into such a subject if made by an ardent teetotaller, though we might at the same time acknowledge his honesty. When statistics refer to questions which allow of differences of opinion we must know the person who makes them and the purpose for which they are to be used before we can fairly appraise their value. Proper account must be taken of the personal factor, which in such statistics is of the first importance. When a number of individuals are engaged in making statistics with respect to matters which are not clearly defined, and which present scope for wide divergencies of opinion, the results of their labours are not comparable.

There is no clear definition of what constitutes a recovery from an attack of mental disease. The director of one hospital for the treatment of mental disorders may consider a man recovered and reckon him to the credit of the statistics of his institution. Another director may look upon such a man as only fit to be at large if he be under proper care and supervision. He therefore discharges him as "relieved," and his statistics suffer thereby. There are other points on which widely differing opinions as to what constitutes a recovery may be held. The absence of a generally accepted definition of a recovery is one great reason why it is absurd to compare the recovery rates in different mental hospitals. The fact that the proportionate number of the cases admitted in whom there is any possibility of recovery may vary to a very large extent is, perhaps, a still greater reason for such comparisons being not only odious but worse than useless. The Report of the Commissioners in Lunacy shows that the number of stated recoveries for the year 1911 in different institutions varies from 20 to 60 per cent. of the admissions. What is the reason for this wide variation? We do not believe that among our mental hospitals of to-day some are so far in advance of others in the skill of their medical staff and the nature of appliances at their command as adequately to account for this marked difference. The explanation lies in the first place in the varying nature of the cases admitted, and in the next place in the uncertain standard as to what constitutes a recovery.

The recovery rate is calculated upon the number of admissions for the year. In county and borough mental hospitals for the period between the years 1869 and 1878 the recovery rate was 35.58 per cent. for men, and 44.33 per cent. for women. Since that period the recovery rate has declined, and for 1911 it was 28.42 per cent. for men and 37.21 per cent. for women. It would be as reasonable, however, to deduce from these figures that the advances made in the knowledge of the causation and course of mental diseases and the attention given to their treatment since 1869 had been of no avail as it would be to come to a similar conclusion because the recovery rate in an institution which stands in the front rank of mental hospitals was last year below the average. The absence of a definition of a recovery does not assist us to find an explanation for this fall in the recovery rate since 1869. The idea as to what was a recovery was probably as vague in 1869 as it is in 1912. When we consider the nature of the material supplied to the physician to work upon, however, we are not surprised at the lowering of the recovery rate, but are rather astonished that it should remain as high as it is. In 1869 there were in all 46,732

certified paupers insane in England and Wales; in 1912 there are 123,400. In 1869 there were of these 28,564 detained in mental hospitals; in 1912 there are 98,889 so detained. In 1869 there were 11,181 looked after in workhouses, etc.; in 1912 there are 19,162 so cared for. In 1869 there were 6,987 living with their friends; in 1912 there are now 5,349 so placed. That is to say that in 1869 for every 2.5 patients sent to a mental hospital 1 was retained in the workhouse; in 1912 for every 5 sent to the hospital 1 was kept in the workhouse. What is more notable is that there are actually considerably fewer insane living at home with their friends to-day than there were in 1869, although the total number has nearly trebled. This means that a considerably larger proportion of incurables are now sent to mental hospitals than was formerly the case. Cases which were kept in the workhouse or with their friends now find their way to the hospital. This is borne out by the common complaint from the heads of mental hospitals as to the large proportion of hopeless cases they receive for treatment. The recovery rate is calculated on the number of admissions, and, as the recoverable cases now bear a smaller proportion to the total number, the recovery rate must necessarily diminish. Whether a large recovery rate be an unmixed good is quite another question.

